# Training Effects and Intelligent Evaluated Pattern of the Holistic Music Educational Approach for Children with Developmental Delay

**DOI:** 10.3390/ijerph181910064

**Published:** 2021-09-25

**Authors:** Liza Lee, Ying-Sing Liu

**Affiliations:** 1 Department of Early Childhood Development and Education, Chaoyang University of Technology, Taichung 413310, Taiwan; lylee@gm.cyut.edu.tw; 2 College of Humanities and Social Sciences, Chaoyang University of Technology, Taichung 413310, Taiwan

**Keywords:** young children, learning effect, intelligent evaluated pattern, HMEAYC, developmentally delayed

## Abstract

This study focuses on the holistic music educational approach for young children developed by music therapists and experts, which combines technology with music, integrates it into the local culture, and frameworks it for holistic education. This method includes 231 children in Taiwan’s early intervention education system for children with developmental delay. The learning occurs twice a week for 40 min over 32 weeks. The results show that implementing the holistic music educational approach can significantly improve children’s ability with developmental delay and that supportive training has a positive effect. In addition, the decision tree explores and develops an intelligently evaluated pattern with highly effective learning. This model has a sensitivity rate of 90.6% on the in-sample, and the comprehensive indicator F is 79.9%, so it has a high reference value. In the future, those involved in education will be able to use the data mining to use the auxiliary decision-making system as an assessment tool for young children participating in education pre- and midterm of the course, to prejudge its continued implementation and learning effectiveness, to decide whether to continue to invest in and adjust the curriculum, and to make more effective use of educational resources.

## 1. Introduction

There have been numerous studies that support the fact that music helps activate the brain [1]. The influence of music on language, movement, and cognitive development of young children has been confirmed by many studies, and it has significant positive auxiliary effects in the early stages of an individual’s development [2]. A study had explored the effects of music on children and found that rhythm and syllabic music sequences had a different impact on their grammar and semantic processing [3]. Children perform better grammatically in regular rhythmic lines than in phonology sequences. Rhythm promotes the perceived and cognitive order required for grammatical processing, while texture encourages the initiation of concepts in language production. Some scholars [4] have studied the impact of music training programs on children’s hearing development and found that children who undergo music training can detect changes in the tone environment. Additionally discovered was that accelerated maturation through cortical auditory processing has the potential to trigger notes, thus supporting the theory that music training in young children can stimulate specific brain changes.

Many studies have also confirmed the improvement of language ability, perception, motor consciousness, cognition, and emotions of children with special needs [5,6]. Scholars [7] propose providing a learning curriculum content and educational environment for young children with different learning needs and argue that without the preparation of the children’s education with disabilities, it may not be possible to realize the idea of learning needs fully. The study [8] found that activity-based interventions or control conditions can increase language expression for preschool children with disabilities.

Scholars [9] used data-driven decision making to help educators identify young children who do not respond and support educators in making online decisions web applications to guide data-driven decision making, which will significantly improve the risk to young children in the language delay. At the same time, children with developmental delay are those preschool children who lag behind their peers in cognitive learning, language communication, movement, interpersonal interaction, and emotional development [10], so less than one to two standard deviations [11] stunt the effects of learning, language, and behavior [12]. Scholars [13] supported implementing early intervention programs to improve the relationship between young children and mild developmental delays and peers. The study [14] supported the early intervention of children with delayed development to participate in the center-based learning modality.

In music education, music knowledge and technical ability are music-centric, and a goal is to develop these skills through voice and musical instrument instruction. Music education was used as embedded teaching to support the cognitive, sensory, and psycho-activity of children. In individual special-needs education programs, the presence of music improves the effectiveness and permanence of their learning [15]. A scholar [16] introduced a holistic perspective and the practice of learning and advanced a concept of supporting the whole-person phenomenon that can be applied to any teaching model within the educational context. Another study [17] combined modern technology with digital music technology and professional teaching methods to provide learning for sick children.

Holistic Music Educational Approach for Young Children (HMEAYC) is based on a music education system developed by Holistic Education [18,19,20,21]. It integrates local culture and uses modern computer technology to combine specialists and music therapists to create an engaging music curriculum model for preschoolers [22]. The system has been extended to music education for local mainstream young children [22]. The use of music therapy for young children with special needs in language and communication, attention, body movements, stable emotions, interpersonal interaction, and other abilities can significantly positively impact both children and the quality of their family life [22].

The social resources and time invested in the implementation of HMEAYC in early childhood special education are enormous. Suppose the researchers can find a method to pre-evaluate the implementation effect of children with developmental delay participating in learning programs. Early understanding of the final learning effectiveness of each learner after the program is implemented. This vital information serves as a reference basis for individual learners to adjust or terminate during the program implementation process and effectively allocate and manage resources through the evaluation model to improve the efficiency of educational resource use. This study was based on the teaching results of children with developmental delay after 32 weeks of HMEAYC, using data mining to establish a model for pre-evaluating the learning effect. Data mining has been widely used in various scientific fields [23,24], and this method can be used to develop intelligent (self-correcting) application systems. The data mining used is based on the decision tree of big data, which can be used to evaluate different models to improve traditional statistical methods [25]. This programming algorithm can be loosened the departed requirements of homogeneity on the research data for the statistic method.

The goals were to understand better which factors will have a more significant impact on the effectiveness of instructional strategies for young children with developmental delay, and midterms assess instructional effectiveness during the implementation of the course. These possible underlying factors include participants’ sex and age, and behavioral capabilities in pre- and midterm of the course are implemented, such as language expressiveness, language comprehension, physical movement, social skills, interpersonal relationships, self-directed, and autonomy. This system provided music educators with a tool for future assessment to improve the efficacy and efficiency of educational resources.

## 2. Data and Empirical Methods

### 2.1. Empirical Data and Definition Variables

The study collected the data of young children with developmental delay, aged between 2 and 6 years, from 2002 to 2019, at an early intervention institute recognized by the Taiwan Fund for Children and Families—Taichung City Child—which collects information to find out whether the participation of preschool young children with developmental delay in HMEEAYC can help improve their learning effectiveness and establish an auxiliary assessment system. The Young Children with Developmental Delay are defined in Article 3, paragraph 11, of the Measures for the Identification of Students with Physical or Mental Disabilities, and Outstanding Talented (Amendment Date: 28 September 2012) was established under Article 16 (2) of the Taiwan Special Education Law. This refers to children under the age of six who, due to physiological, psychological, or social environmental factors, are significantly delayed in the development of perception, cognition, movement, communication, social emotions, or self-care abilities than those of the same age, and whose types of disabilities cannot be determined. Multi-evaluation is used to identify children with developmental delay, standardized assessments, direct observations, interviews, medical examinations, etc. (Art. 2 of the Measures for the Identification of Students with Physical or Mental Disabilities and Outstanding Talented qualifications) adapted according to the individual situation. Finally, the government will issue a handbook (certificate) for disabilities and record case information for future judgment. The study sampling is based on the children with developmental delay identified in the physical and mental disabilities handbook as the research subjects.

The study pre-recorded 244 young children with developmental delay who took part in the course, excluding 13 people who were incomplete without authorized consent and recorded images. A sample of 231 children with developmental delay participated in the HMEAYC effectiveness evaluation learning and study. The direct instruction component was implemented with twice weekly sessions for 40 min each over 32 weeks. The collection and data repository is the responsibility of two observers, who must be qualified after participating in Taiwan’s professional course training for two assessment tools, Social Behavior Assessment System for Preschool (SBASP) [26] and Adaptive Behavior Assessment System-II (ABAS-II) [27]. In observing instructional delivery, attention was given to avoid interfering with the participants in their instruction and reduce experimental errors; data were collected by non-participant observation using a video-recording method cross-compared with the data from at least two professional observers. In addition, during video observation, when the two observers had an inconsistent view of the analysis of the video footage, and further explanations and concerns about observational behavior are needed, and the teacher or professional therapist who was teaching at the time was consulted in the hope of improving the accuracy of the login data with multiple verifications. All video archives and verified materials in this study were eventually kept in the Center of Holistic Music for Young Children in Taichung, Taiwan.

The primary reference, in terms of effective evaluated indicators [19,21], were divided into the following: (1) linguistic competence consists of language expressiveness (code: LE) and language comprehension (code: LC); (2) physical ability consists of physical movement (Code: PM); (3) interaction with people consists of social skills (code: SS) and interpersonal relationships (code: IR); (4) self-exposure consists of self-directedness (code: SD) and autonomy (code: AB). The language comprehension (LC) (7 questions), interpersonal relationships (IR) (8 questions), and autonomy (AB) (8 questions) are based on the Social Behavior Assessment System for Preschool (SBASP) [26]. The language expressiveness (LE) (25 questions), social skills (SS) (24 questions), physical movement (PM) (27 questions), and self-directedness (SD) (25 questions) are based on the Adaptive Behavior Assessment System-II *(ABAS-II)* [27]. Each question has five scores. The higher the score, the better the evaluation result. In the assessment of learning effectiveness, the pre-value (code: pre) is measured, which was attached for events before the implementation of the HMEAYC, the mid-value (code: mid) was applied after the eighth week, and the post-value (code: post) was used after the conclusion of the entire 32 weeks.

Based on the comparison of the pre-values and post-values of the evaluated effectiveness indicator in the items mentioned above, a function that measures the improvement of the learning ability of a single item is:

(1) $$P_{i}\left(X_{\mathrm{post},i},X_{\mathrm{pre},i}\right)=\{\begin{array}{c} 1\;,\;\;\;X_{\mathrm{post},i}>X_{\mathrm{pre},i}\;\\ 0,\;\;\;\;\;\;\;\;\;\;\mathrm{otherwise} \end{array},$$

In Equation (1), i can be represented as LE, LC, PM, SS, IR, SD, and AB, respectively, seven effectiveness evaluated indicators, and $X_{\mathrm{post},i}$ and $X_{\mathrm{pre},i}$ represent the post-value and pre-value of a single indicator for i. Thus, the number of evaluated indicators that can be improved is $N_{\mathrm{II}}=\sum P_{i}$, and a function of high-impact ability for improvement is defined as:

(2) $$\mathrm{ICI}\left(N_{\mathrm{II}}\right)=\{\begin{array}{c} 1\;,\;\;\;\;\;\;N_{\mathrm{II}}\geq 4\;\\ 0,\;\mathrm{otherwise} \end{array},$$

In Equation (2), $\mathrm{ICI}\left(N_{\mathrm{II}}\right)$ is a measure after the implementation of HMEAYC for 32 weeks, and for participants in the seven evaluated indicators, there will be at least four indicators with a post-value higher than the pre-value ($N_{\mathrm{II}}\geq 4$), at this time, given $\mathrm{ICI}\left(N_{\mathrm{II}}\right)=1$; otherwise, the value is 0.

The midterm calculation of the indicators changed after the 8th week of the implementation of HMEAYC are ($X_{\mathrm{mid},i}$ − $X_{\mathrm{post},i}$)/$X_{\mathrm{post},i}$, so that the mid-changes in the seven evaluated indicators can be calculated separately for language expressiveness (code: LEMC), language understanding (code: LCMC), physical movement (code: PMMC), social skills (code: SSMC), interpersonal relationships (code: IRMC), self-directed (code: SDMC) ,and autonomy (code: AB), respectively. The definition, code, and illustrations of the study variables are combined in Table 1.

### 2.2. HMEAYC

Holistic Music Educational Approach for Young Children (HMEAYC) is an educational training model developed by the Research Center of Holistic Development for Young Children and a music activity curriculum designed specifically for the development of preschool children in Taiwan. It covers educational perspectives, such as education, sociology, and philosophy, combining music education and music therapy and integrates them into a curriculum model, with spindles applied to the cross-cutting integration of education and treatment for preschool children and children with special needs [18,19]. This set of course music materials is a combination of traditional musical instruments, technical instruments, and multi-sensory devices, such as iPads [28] and Soundbeam [22,29,30]. Hardware equipment and teaching methods [19]: (1). Multi-sensory room includes Korner Kurve Ball Pool, Talking Cube, Circular Set of Musical Music Lights, Musical Jumping Pad, Star Master Projector Light Lamp, Sound Bed, Laser Lights, Soundbeam 5, and virtual interactive games. (2). Figurenotes [20]. The purpose of the curriculum design is to expect to teach will benefit the overall children’s development. The curriculum content includes Hello Song, Attendance Song, Musical Games, Musical Storytelling, Relaxation Time, and Goodbye Song [19].

### 2.3. The Decision Tree

In order to obtain a preliminary look at whether the evaluation indicators differ significantly after receiving the implementation of HMEAYC before the decision tree analysis, statistical inferences are made using t-tests (paired value for post minus pre) and the Wilcoxon matched-pairs signed-ranks tests. Although these two statistic tests have many limitations in terms of methods and samples. For example, the assumption is that: (1) individual children with developmental delay are not different in their sensitivity to the evaluation indicators during training; (2) t-statistics assume that the average of the paired difference values for each observation follows a normal distribution based on the central limit theorem (CLT), and that the variance is homogeneous; (3) the Wilcoxon method relaxes the restrictions in situations where the population is unknown and non-normal distribution occurs. This study focuses on using decision trees to assess the classification of the factors influencing the indicators, and the results of these two tests are not strictly correct and non-necessary. The primary purpose is to increase more information about the sample before the tree structure model is established and serves as an additional reference to support the adoption of decision trees for further analysis. Therefore, the two statistical tests in this study infer that there will be significant differences between the evaluation indicators’ post-measurement value and the pre-measurement value. The measurement values as observational evaluation indicators will change significantly after the implementation of HMEAYC as a preliminary judgment of the existence of implementation effect.

The decision tree belongs to an algorithm of supervised learning, classified by specific rules to establish a tree structure, which are inductive, and extract the rules for predicting unknown samples. In a based tree structure, the layer’s design illustrates the effect of the arguments of each layer on the dependent variable (the target variable). When the dependent variable is the categorical form, the decision tree is established as a classification tree. In this study, the classification and regression trees (CART) [21,31] apply the Gini index as the criterion for determining branching variables and classifying the data in each branch node to establish a structure of the decision tree with binary classification.

The accuracy can measure indicators for the evaluation tree pattern, sensitivity, precision, and comprehensive indicator F. At this point, it is assumed that the learning dataset will be classified into two categories, good (where the learning effect is better) and bad (where the learning effect is not better), and that if the data are predicted and consistent with the actual situation, there are “true good” and “true bad.” Where the forecast is inconsistent with the actual situation, there are “false good” and “false bad,” so you can obtain an accuracy equal to (true good + true bad)⁄(total sample), sensitivity equal to (true good)⁄(true good + false bad), precision equal to (true good)⁄(true good + false good), and the composite indicators F equal to (2 × precision × sensitivity)⁄(precision + sensitivity). In four indicators, the higher value of the indicator indicates that the lower the misjudgment rate of the tree pattern, the more accurate predictive power there is, especially in the section of concern classified as good (effect learning is better), where the higher the sensitivity is, the result is more expected in the study.

## 3. Empirical Results and Analysis

The sample size in this study was 231, 148 males accounted for 64.07 % of the sample, and 83 females accounted for 35.93% of the sample. Ages ranged from 24 months to 71 months, and the mean ± standard deviation was 49.03 ± 14.22 months. Table 2 shows the mean ± standard deviation of the seven evaluation indicators, namely, language expressiveness, language comprehension, physical movement, social skills, interpersonal relationships, self-directed, and autonomy, and the measured pre-values of prior to the course implementation, mid-values, and pre-to-mid changes after implementation of 8 weeks, post-values and pre-to-post changes after the 32 weeks of implementation, respectively. In the results in Table 2, the seven evaluated indicators found that the mean of post-value was more than the mean of pre-value, and the mean of the pre-to-post changes was positive. Among them, the mean of the pre-to-post changes in interpersonal relationship, autonomy, and language expressiveness was higher at 0.16, 0.09, and 0.06, respectively, showing an increase of 15.74%, 8.75%, and 5.79%, respectively, after the implementation of the HMEAYC’s 32-week course. 

Table 3 shows the results of the difference in means *t*-tests (paired value for post minus pre) and the Wilcoxon matched-pairs signed-ranks tests. Table 3 shows the results of *t*-tests; at a 5% significant level, it can be determined that the mean of the paired difference is significantly positive (*p*-values less than 0.05) for the seven evaluated indicators, respectively. Therefore, it shows that after 32 weeks of HMEAYC implementation, the seven evaluation indicators’ post-value was more significant than the pre-value. This supports the fact that the implementation of HMEAYC instruction for young children with developmental delay in language expressiveness, language comprehension, physical movement, social skills, interpersonal relationships, self-directed, and autonomy had a significantly positive effect. Overall, results indicate that HMEAYC had a positive impact on the needs of children with developmental delay. In addition, a further comparison of the results of the non-parametric statistical test’s Wilcox on matched-pairs signed-ranks tests found that the results are roughly the same as those of the *t*-tests. 

These results have been confirmed in previous studies [19,20], but those must be limited to conditions where the data are not heterogeneous. In addition, statistical tests can only obtain information on the average level of the overall sample on individual evaluation indicators. It is impossible to understand what primary factors or mid-period learning conditions will affect the final learning effects for the individual learner. Therefore, a decision tree will be used for data mining to determine which factors will be better for children with developmental delay after implementing the 32-week HMEAYC program and finding out a set of classification rules.

Table 4 shows the number and accounting for proportion of samples based on the post-value more than the pre-value, in the seven evaluated indicators, respectively. Additionally, it was found that the number of persons who conform to a condition fell between 93 and 161 people (sample size is 231) in each indicator. The percentage of SD that can be improved is up to 69.70%, with the remaining six indicators falling to between 40% and 60%, which indicates the implementation of a 32-week HMEAYC course, although it had a significant positive impact on developing toddlers. However, most of the indicators can be improved by about half of the sample size. Hence, future attention will be paid to the reasons that affect more indicators and trying to capture which factors will have a better impact.

**Figure 1:**
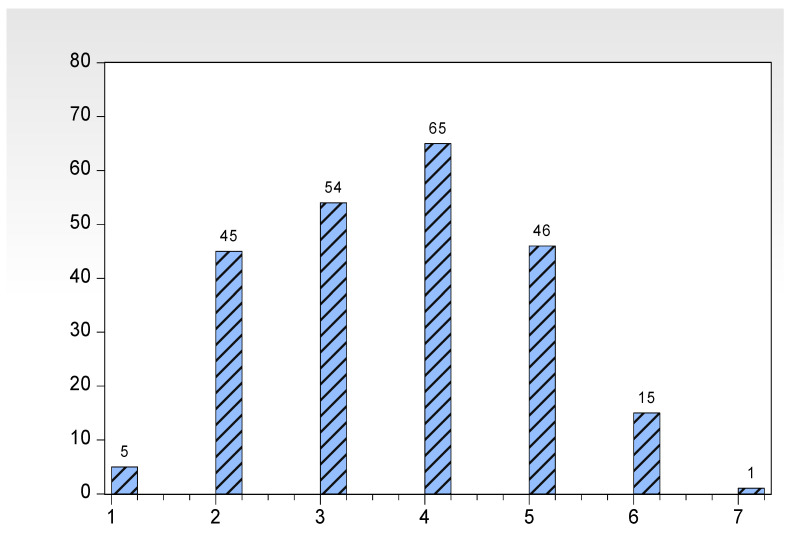
The frequency distribution charts on the items of effective impact evaluated indicators after the implementation of HMEAYC.

Figure 1 shows the distribution charts for the number ($N_{\mathrm{II}}$) of evaluated indicators of a good impact after the implementation of HMEAYC. In Figure 1, the sample N = 231, shows that there will be at least one or more evaluated indicators in the post-value more than pre-value, where the number ($N_{\mathrm{II}}$) of evaluated indicator of good impact is distributed between 1 and 7. At $N_{\mathrm{II}}\;$= 4, there were 65 people in a maximum group, compared with 104 people for 1 ≤$\;N_{\mathrm{II}\;}$< 4, with a mean ± standard deviation of 3.6537 ± 1.2620. The implementation of HMEAYC must be time-consuming and use a considerable amount of educational resources, so that this study will use the participants’ data, based on the decision tree, the pre-values of the eighth week, to develop a system that will assess the implementation of HMEAYC in advance, which will have a high impact on the participants. Using a function of the high-impact improvement ability such as $\mathrm{ICI}\left(N_{\mathrm{II}}\right)$ as a dependent variable concerns the classification of the decision tree in the case of $\mathrm{ICI}\left(N_{\mathrm{II}}\right)$ = 1, that is, after implementing HMEAYC for 32 weeks, the participants have the good learning effects of the high impact of the decision tree classification.

**Figure 2:**
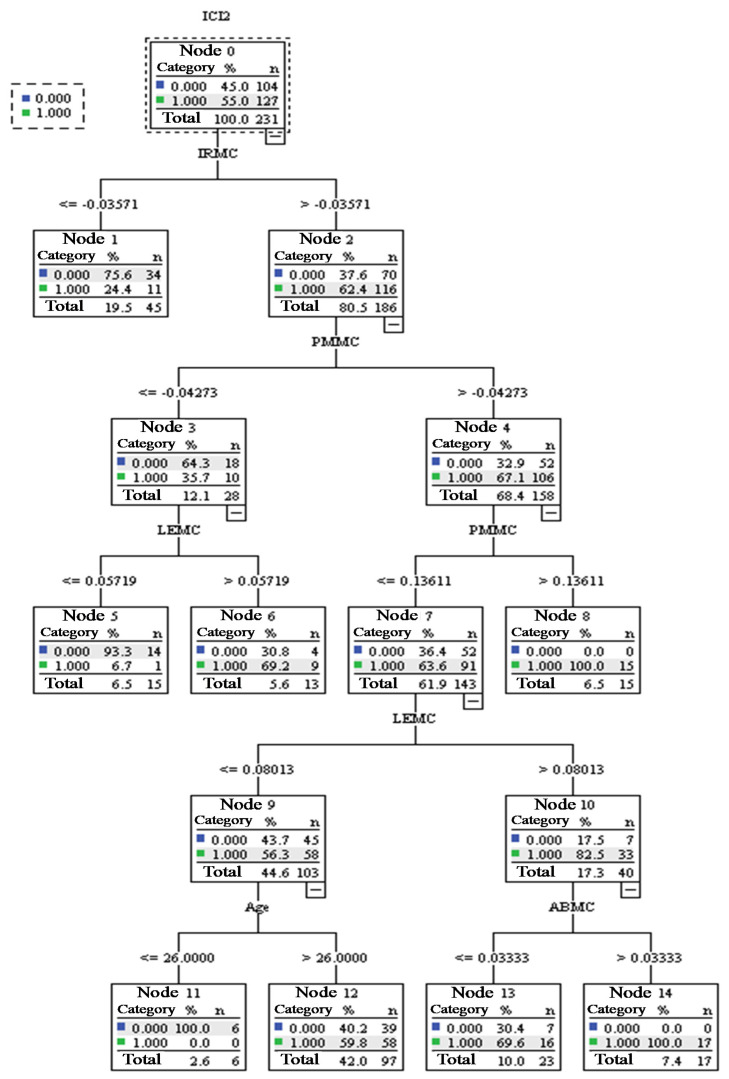
A tree structure pattern that has a better learning effect on the sample after the implementation of the HMEAYC.

Figure 2 shows the sample number of 231 people, using the CRT growth method obtained by the decision tree pattern. The growth condition of the tree is 5 in the maximum depth, the minimum number in the parent node is 20, and the minimum number in the child node is 5. The pattern has a risk value of 0.27, a standard error of 0.03. In $\mathrm{ICI}\left(N_{\mathrm{II}}\right)\;$= 1 is a sensitivity of 83.40% (precision of 71.4%), 73.2% of accuracy, the comprehensive indicator F is 79.9%, and there are 15 nodes. There are 15 independent variables in the following order: IRMC, IRpre, PMMC, ABMC, SDpre, SDMC, LEMC, SSMC, PMpre, LEpre, LCMC, age, ABpre, SSpre, and LCpre. The importance of the independent variables is in the following order: IRMC (0.06), LEMC (0.05), PMMC (0.04), LCMC (0.03), ABMC (0.02), age (0.02), IRpre (0.02), LEpre (0.01), and PMpre (0.01). In addition, the gender differences are not related to a dependent variable.

In Node 0, the sample of 231 people, $\mathrm{ICI}\left(N_{\mathrm{II}}\right)\;$= 1, has 127 people accounting for 55.0% of the sample. The branches below Node 0 are Node 1 and Node 2 (First layer). In Node 1 (IRMC ≤ −0.04; 45 people accounted for 19.5% of the sample), 11 participants of the high-impact learning effect ($\mathrm{ICI}\left(N_{\mathrm{II}}\right)\;$= 1) accounted for 24.4% of the total number of Node 1; therefore, when IRMC≤−0.04 is shown, 75.6% of the participants will have a limited learning effect ($N_{\mathrm{II}}\;$≤ 3). In Node 2 (IRMC > −0.04; 186 people accounted for 80.5% of the sample), 116 participants of the high-impact learning effect ($\mathrm{ICI}\left(N_{\mathrm{II}}\right)\;$= 1) accounted for 62.4% of the total number of Node 2; this proportion is much higher than the 24.4% at Node 1. Branching down from Node 2 into Node 3 (PMMC ≤ −0.04; 28 people accounted for 12.1% of the sample) and Node 4 (PMMC > −0.04; 158 people accounted for 68.4% of the sample) forms the second layer. The following is a description of Node 3 and Node 4.

1. In Node 3, 10 participants of the high-impact learning effect ($\mathrm{ICI}\left(N_{\mathrm{II}}\right)\;$= 1) accounted for 35.7% of the total number of Node 3. Branching down from Node 3 into Node 5 (LEMC ≤ 0.06; 15 people) and Node 6 (LEMC > 0.06; 13 people) forms the third layer. In Node 5, when LEMC ≤ 0.06, only one person is $\mathrm{ICI}\left(N_{\mathrm{II}}\right)\;$= 1, accounting for 6.7% of the total number of Node 5, showing that when IRMC > −0.04, PMMC ≤ −0.04, and LEMC ≤ 0.06, there will be up to 92.3% of the participants with a high ratio of limited effect ($N_{\mathrm{II}\;}\;$≤ 3). In addition, in Node 6, when LEMC > 0.06, nine people are $\mathrm{ICI}\left(N_{\mathrm{II}}\right)\;$= 1, accounting for 69.2% of the total number of Node 6, indicating that when IRMC > −0.04, PMMC≤−0.04, and LEMC>0.06, 69.2% of the participants will have the high-impact learning effect ($\mathrm{ICI}\left(N_{\mathrm{II}}\right)\;$= 1).
2. In Node 4, 106 participants of the high-impact learning effect ($\mathrm{ICI}\left(N_{\mathrm{II}}\right)\;$= 1) accounted for 67.1% of the total number of Node 4, indicating that when IRMC > −0.03571 and PMMC > −0.04, 67.1% of the participants will have the high-impact learning effect ($\mathrm{ICI}\left(N_{\mathrm{II}}\right)\;$= 1). Branching down from Node 4 into Node 7 (PMMC ≤ 0.14; 143 people accounted for 61.9% of the sample) and Node 8 PMMC >0.14; 15 people) forms the third layer. In Node 8, 15 people are $\mathrm{ICI}\left(N_{\mathrm{II}}\right)\;$= 1, so 15 people account for 100.0% of the total number of Node 8, showing that when IRMC > −0.04 and PMMC > 0.14, they will have the high-impact learning effect ($\mathrm{ICI}\left(N_{\mathrm{II}}\right)\;$= 1). Besides, in Node 7, when PMMC ≤ 0.14, there are 91 people as $\mathrm{ICI}\left(N_{\mathrm{II}}\right)\;$= 1, accounting for 63.6% of the total number of Node 7, indicating that when IRMC > −0.04 and −0.04 < PMMC ≤ 0.14, 63.6% of the participants will have the high-impact learning effect. Branching down from Node 7 into Node 9 (LEMC ≤ 0.08; 103 people) and Node 10 (LEMC > 0.08; 40 people) forms the fourth layer. The following is a description of Node 9 and Node 10.

- In Node 9, 58 people are $\mathrm{ICI}\left(N_{\mathrm{II}}\right)\;$= 1, accounting for 56.3% of the total number of Node 9. Branching down from Node 9 into Node 11 (age ≤ 26; 6 people) and Node 12 (age > 26; 97 people) forms the fifth layer. In addition, in Node 11, when age ≤ 26, 0 people is $\mathrm{ICI}\left(N_{\mathrm{II}}\right)\;$= 1, accounting for 0.0% of the total number of Node 11, indicating that when IRMC > −0.04, −0.04 < PMMC ≤ 0.14, LEMC ≤ 0.08, and age ≤ 26, all participants would not have the high-impact learning effect. This result indicated that when participants were too young, the learning did not perform well. In Node 12, when age > 26, 58 people are $\mathrm{ICI}\left(N_{\mathrm{II}}\right)\;$= 1, accounting for 59.8% of the total number of Node 12.
- In Node 10, 33 people are $\mathrm{ICI}\left(N_{\mathrm{II}}\right)\;$= 1, accounting for 82.5% of the total number of Node 10, indicating that the participants of high ratio will have a high-impact learning effect. Branching down from Node 10 into Node 13 (ABMC ≤ 0.03; 23 people) and Node 14 (ABMC > 0.03; 17 people) forms the fifth layer. The following is a description of Node 9 and Node 10. In addition, in Node 13, when ABMC ≤ 0.03, 16 people are $\mathrm{ICI}\left(N_{\mathrm{II}}\right)\;$= 1, accounting for 69.6% of the total number of Node 13. In Node 14, when ABMC > 0.03, 17 people are $\mathrm{ICI}\left(N_{\mathrm{II}}\right)\;$= 1, accounting for 100.0% of the total number of Node 14, indicating that when IRMC > 0.04, −0.04 < PMMC ≤ 0.14, LEMC > 0.08, and ABMC > 0.03, all participants would have the high-impact effect upon learning.

## 4. Discussion and Conclusions

### 4.1. Discussion

This study explores the impact of HMEAYC on the needs of children with developmental delay and the effectiveness of implementing a system for high-impact instruction. Data from this study confirm that the HMEAYC showed a significant improvement in indicator values, such as language expressiveness, language comprehension, physical movement, social skills, interpersonal relationships, self-directedness, and autonomy for children with developmental delay. The result was consistent with past studies [18,19,20,21,22,28,29,30]. This study supports past studies on implementing the HMEEAYC curriculum for young children with developmental delay in Taiwan. This course will improve most young children’s ability in language expressiveness, language comprehension, physical movement, social skills, interpersonal relationships, self-directed, and autonomy.

In the long run, supporting the implementation of HMEAYC had a significantly positive effect on the learning of children with developmental delay and suggested that HMEAYC should be extended and promoted in the early treatment education of special-needs children. On top of that, this study also developed and implemented an intelligent evaluation system to increase learning effectiveness. This system evaluates the learner’s final learning effectiveness in advance during the teaching process as a reference for the education resource manager to decide whether to allow the learner to continue participating in the HMEAYC course. The advantage of this auxiliary assessment system is that learners with poor learning outcomes can be pre-screened, and those who provide management education resources can decide whether to adjust. Ensure that the use of this set of high-cost educational resources of music courses for each learner to obtain a high learning effect to use educational resources effectively.

### 4.2. Conclusions

Using the study protocols, which were established in advance, after implementing HMEAYC of developmentally delayed in young children, especially those with better learning results, with up to 90.6% of the sensitivity and the comprehensive F index is 79.9%. Consequently, the researchers considered it to have reference value and worthy of further extending the promotion. Finally, it is found that the learning effect of the first eight weeks has essential reference value in the developed pattern, especially where changes in the interpersonal relationships and physical movement are significant for the better learning effects on the first eight weeks. It is recommended that the technology of decision trees may be considered for continuous extension in special early childhood education in the future.

The limitations of this study are (1). Missing control group: This study emphasizes that each participant’s learning ability has been changed in the vertical section. The control conditions for the cross-section are temporarily ignored based on the difficulty of sample collection and the algorithm’s promise of the sample size. (2). Ignore the impact of subsequent additions on the currently established auxiliary assessment model: The auxiliary assessment system established by the Institute will change in the future with the data of the new participants, and the current results will be based on existing samples. (3). Local restrictions on sample collection: The study is currently limited to samples of young children with developmental delay in one region of Taiwan to establish an evaluation model. In the future, children with disabilities who are enrolled in other neighboring regions or countries, as well as other conditions, will be further expanded.

## Figures and Tables

**Table 1:** The code, type, definition, and description of the study variables.

Study Variables	Code	Type: Scale	Definition and Description
Improvement Capacity Indicators	ICI	Category	A given ICI was 1 when at least four evaluated indicators were more post-value than pre-value; otherwise, the given ICI is 0.
Sex	Sex	Category	Boy and girl
Age	Month	Order	The age ranges from 24 months to 72 months
Language Expressiveness	LE	Measure	pre-value: LEpre; mid-value: LEmid; post-value: LEpost; mid-change: LEMC
Language Comprehension	LC	Measure	pre-value: LCpre; mid-value: LCmid; post-value: LCpost; mid-change: LCMC
Physical Movement	PM	Measure	pre-value: PMpre; mid-value: PMmid; post-value: PMpost; mid-change: PMMC
Social Skills	SS	Measure	pre-value: SSpre; mid-value: SSmid; post-value: SSpost; mid-change: SSMC
Interpersonal Relationships	IR	Measure	pre-value: IRpre; mid-value: IRmid; post-value: IRpost; mid-change: IRMC
Self-Directed	SD	Measure	pre-value: SDpre; mid-value: SDmid; post-value: SDpost; mid-change: SDMC
Autonomy	AB	Measure	pre-value: ABpre; mid-value: ABmid; post-value: ABpost; mid-change: ABMC

**Table 2:** The measured variables for the descriptive statistics (N=231).

Variable	Pre-Value	Mid-Value	Post-Value	Mid-Change	Post-Change
Estimator	Mean ± Std. Dev	Mean ± Std. Dev	Mean ± Std. Dev	Mean ± Std. Dev	Mean ± Std. Dev
Language Expressiveness	15.03 ± 3.33	15.06 ± 3.75	15.67 ± 4.37	0.01 ± 0.19	0.06 ± 0.28
Language Comprehension	12.15 ± 2.73	13.04 ± 3.39	12.69 ± 3.37	0.08 ± 0.20	0.04 ± 0.16
Physical Movement	29.50 ± 9.66	29.87 ± 9.50	30.36 ± 9.08	0.02 ± 0.07	0.04 ± 0.11
Social Skills	22.87 ± 6.28	23.24 ± 6.46	23.72 ± 6.61	0.02 ± 0.10	0.04 ± 0.14
Interpersonal Relationships	9.48 ± 2.14	10.00 ± 2.27	10.52 ± 1.61	0.08 ± 0.28	0.16 ± 0.27
Self-Directed	33.50 ± 6.21	33.87 ± 6.32	34.89 ± 5.97	0.01 ± 0.05	0.05 ± 0.08
Autonomy	10.09 ± 1.79	10.46 ± 1.83	10.90 ± 2.38	0.05 ± 0.13	0.09 ± 0.19

**Table 3:** The tests of the differences between the paired samples in mean (N = 231).

No	Paired Sample	Difference		t-Tests		Wilcoxon Matched-Pairs Signed-Ranks Tests	
		Mean	Std. Dev	t-Stat.	*p*-Value (Two-Tailed)	z-Stat.	*p*-Value (Two-Tailed)
1	LEpost-LEpre	0.63 **	3.79	2.53	0.01	−1.94 ^b^	0.05
2	LCpost-LCpre	0.54 ***	1.79	4.59	<0.001	−4.04 ^b^	<0.001
3	PMpost-PMpre	0.86 ***	2.85	4.58	<0.001	−4.91 ^b^	<0.001
4	SSpost-SSpre	0.86 ***	3.05	4.28	<0.001	−4.69 ^b^	<0.001
5	IRpost-IRpre	1.05 ***	2.30	6.94	<0.001	−7.12 ^b^	<0.001
6	SDpost-SDpre	1.39 ***	2.31	9.15	<0.001	−9.54 ^b^	<0.001
7	ABpost-ABpre	0.81 ***	1.95	6.27	<0.001	−6.82 ^b^	<0.001

Note: *** and ** represent significance at the 1% and 5% levels; ^b^ is based on the negative ranked.

**Table 4:** The counts in the evaluation indicator, based on the post-value more than the pre-value, and its proportion to the sample (N = 231).

Terms	LE	LC	PM	SS	IR	SD	AB
Counts	96	93	119	119	131	161	125
%	41.56	40.26	51.52	51.52	56.71	69.70	54.11

## Data Availability

Data sharing is not applicable to this article.
